# Prevalence of Borderline Oxacillin-Resistant *Staphylococcus aureus* Strains (BORSA) and Patients’ Clinical Outcome in A Tertiary Teaching Hospital in East Coast Malaysia

**DOI:** 10.21315/mjms-03-2026-184

**Published:** 2026-08-31

**Authors:** Wil Sern Lim, Siti Hafsyah Mohd Hariri, Zakuan Zainy Deris, Wei Chuan Chua, Wan Alif Syazwani Wan Alias, Nur Syafiqah Mohamad Nasir, Siti Asma’ Hassan

**Affiliations:** 1 Department of Medical Microbiology and Parasitology, School of Medical Sciences, Health Campus, Universiti Sains Malaysia, 16150 Kubang Kerian, Kelantan, Malaysia; 2 Hospital Pakar Universiti Sains Malaysia, Health Campus, Universiti Sains Malaysia, 16150 Kubang Kerian, Kelantan, Malaysia

**Keywords:** antimicrobial resistance, BORSA, mecA gene, nuc gene, Staphylococcus aureus

## Abstract

**Background:**

Borderline Oxacillin-Resistant *Staphylococcus aureus* (BORSA) is an emerging *S. aureus* strain that exhibits reduced susceptibility to penicillinase-stable penicillins (PRPs) with oxacillin MICs between 2 and 4 μg/mL. This resistance is attributed to *blaZ* hyperproduction or point mutations in penicillin-binding proteins. Their MIC pattern makes clinical management of infected patients challenging. This study aims to estimate the prevalence of BORSA and describe patient clinical outcomes based on prescribed antibiotic regimens.

**Methods:**

A total of 307 archived isolates collected over six months from June to December 2024 from clinical cases of *S. aureus* were screened using cefoxitin and oxacillin disc diffusion. Potential BORSA isolates were confirmed by gradient diffusion testing. Nitrocefin and double-disc synergy tests confirmed β-lactamase production. Patients’ clinical and demographic data were retrospectively reviewed and analysed for outcomes according to the prescribed antibiotic regimen.

**Results:**

The prevalence of BORSA was 6.5% (*n* = 20). All isolates exhibited β-lactamase production. Most isolates (65%, *n* = 13) were recovered from bloodstream infections, predominantly affecting adults (mean age 48.5 years) with comorbidities, specifically diabetes mellitus (65%, *n* = 13) and renal pathologies (45%, *n* = 9). Resistance to tetracycline and fusidic acid was observed in 65% (*n* = 13) and 25% (*n* = 5) of isolates, respectively. Patients managed with β-lactam-based regimens achieved clinical resolution, whereas treatment with vancomycin resulted in a 50% mortality rate.

**Conclusion:**

The prevalence of BORSA at our hospital was 6.5%. Phenotypic evidence suggests this resistance is driven by β-lactamase hyperproduction. Accurate identification is clinically critical to avoid unnecessary use of vancomycin or therapeutic failure with standard penicillin.

## Introduction

Antimicrobial resistance among staphylococci remains a persistent obstacle to effective infection management worldwide. Within this evolving landscape, *Staphylococcus aureus* (*S. aureus*) has emerged as a pathogen of particular concern due to its considerable genetic adaptability. Although primarily a commensal inhabitant of the anterior nares, skin, and mucosal niches in humans and mammalian hosts, *S. aureus* possesses significant pathogenic potential. It is the aetiological agent of a diverse spectrum of clinical conditions, ranging from superficial skin and soft tissue infections to life-threatening invasive pathologies, such as bacteraemia, infective endocarditis, necrotising pneumonia, and osteomyelitis, all of which contribute to substantial morbidity and mortality (1).

Methicillin-resistant *S. aureus* (MRSA) constitutes a persistent and critical public health burden. The resistance phenotype is predominantly mediated by the acquisition of the *mecA* gene or its structural homologs (e.g., *mecC*) (2). These genetic determinants encode alternative penicillin-binding proteins (PBPs), specifically PBP2a or PBP2c, which are characterised by a significantly reduced binding affinity for β-lactam antibiotics (3). This low-affinity interaction permits continued cell wall synthesis even in the presence of high antibiotic concentrations, effectively nullifying the therapeutic efficacy of the drug class (4).

Borderline Oxacillin-Resistant *S. aureus* (BORSA) represents a distinct phenotypic subset exhibiting reduced susceptibility to penicillinase-resistant penicillins (PRPs). In contrast to classical MRSA, BORSA isolates are genotypically defined by the absence of *mecA* and *mecC* determinants and consequently lack the alternative PBP2a expression. The resistance phenotype in BORSA is primarily attributed to the hyperproduction of staphylococcal β-lactamase (encoded by the *blaZ* gene) (5), which hydrolyses the antibiotic effectively despite the stability of PRPs. Alternative resistance mechanisms have also been postulated, including the acquisition of novel plasmid-mediated β-lactamases or structural alterations (point mutations) in native PBPs that reduce antibiotic binding affinity (6).

Currently, a universally standardised consensus regarding the precise phenotypic definition of BORSA remains elusive. Proposed diagnostic thresholds for oxacillin Minimum Inhibitory Concentrations (MICs) exhibit significant heterogeneity across studies, ranging from 1 to 8 μg/mL. Despite this variability, the prevailing convention within contemporary literature categorises *S. aureus* isolates exhibiting oxacillin MICs between 2 and 4 μg/mL as the borderline resistant phenotype (7). There is a paucity of standardised clinical guidelines governing the therapeutic management of BORSA infections. Despite the elevated MICs observed in vitro, several investigators hypothesise that the BORSA phenotype does not necessarily translate to in vivo therapeutic failure (8). Consequently, it is postulated that these infections may remain amenable to treatment with PRPs, such as cloxacillin, provided that appropriate dosing regimens are utilised to maintain serum concentrations above the MIC (9).

This study addresses the gap in the local prevalence of BORSA among clinical *S. aureus* isolates. Concurrently, the research will investigate the clinical outcomes of patients infected with BORSA strains, specifically categorising patient progress based on the type of antibiotic therapy prescribed by their attending physicians. These findings establish a baseline BORSA prevalence for this tertiary hospital that can inform local antibiotic susceptibility testing protocols and support future surveillance of resistance trends.

## Methods

This is a cross-sectional study involving a total of 307 *S. aureus* archived isolates collected over six months, from June 2024 to December 2024, from the Microbiology laboratory. Isolates obtained from MRSA screening swabs, as well as duplicate isolates from the same patient during a single episode of infection, were excluded. The detailed screening, MIC determination, and molecular confirmation workflow is illustrated in Figure 1. Species identification was performed using MALDI-TOF MS (Bruker Daltonik GmbH, Bremen, Germany). Subsequently, antimicrobial susceptibility testing (AST) was conducted on 307 S. aureus isolates using the Kirby-Bauer disc diffusion method on Mueller-Hinton agar (Thermo Scientific Microbiology Sdn. Bhd., Malaysia), in accordance with the CLSI M100 34th edition guidelines (10). Antibiotics tested included cefoxitin (30 μg) and oxacillin (1 μg) (BMS Diagnostics, Malaysia), as well as erythromycin (15 μg), fusidic acid (10 μg), clindamycin (2 μg), rifampin (5 μg), linezolid (30 μg), trimethoprim-sulfamethoxazole (1.25/23.75 μg), penicillin (10 units), and tetracycline (30 μg) (Bioanalyse, Türkiye). All plates were incubated at 35 ± 2 °C in ambient air for 24 h; zone diameters were then determined using an automated zone reader with manual verification. Isolates susceptible to cefoxitin (zone diameter ≥ 22 mm) were further evaluated using oxacillin disc diffusion; zones were interpreted according to archived 2007 CLSI breakpoints (11), whereby ≥ 13 mm is susceptible, 11 to 12 mm intermediate, and ≤ 10 mm resistant. The utilisation of these historical criteria was necessitated by the shift in current guidelines. This is because CLSI no longer recommends using the 1 μg oxacillin disc diffusion test for human *S. aureus* isolates, as it has been largely superseded by the highly reliable 30 μg cefoxitin disc diffusion screen or direct mecA gene testing. Strains with oxacillin zone diameters ≤ 12 mm underwent MIC determination using gradient diffusion strips (Liofilchem, Italy). For blood culture isolates initially processed via the VITEK^®^ 2 system (bioMérieux, France), the system’s software automatically flagged isolates as “potential BORSA” if they displayed a negative cefoxitin screen paired with an oxacillin MIC ≥ 4 μg/mL. All such flagged isolates were then subjected to manual confirmation using gradient diffusion MIC strips.

Statistical analysis was performed using IBM SPSS Statistics version 29. Categorical variables were summarised as frequencies and percentages. Associations between clinical characteristics and patient outcomes were assessed using Fisher’s exact test because of the small sample size and sparse cell counts. A two-sided *P*-value < 0.05 was considered statistically significant.

### Operational Definition

BORSA was definitively defined as *S. aureus* exhibiting an oxacillin MIC ≥ 2 μg/mL upon manual gradient diffusion strip testing after 24 h of incubation at 35 ± 2 °C, in accordance with established epidemiological cut-off values (12).

Oxacillin resistance was evaluated using gradient diffusion strips on 4% NaCl screening agar (Isolab Sdn. Bhd., Malaysia) to induce phenotypic expression (10). β-lactamase activity was screened using nitrocefin discs (Isolab Sdn. Bhd., Malaysia). Screening for hyperproduction of β-lactamase was performed on 4% NaCl Mueller-Hinton agar by placing an oxacillin (1 μg) disc 20 mm (centre-to-centre) from an amoxicillin-clavulanate (20/10 μg) disc (13). The presence of a synergistic “keyhole” phenomenon was recorded following incubation. The antimicrobial susceptibility profile of the 20 confirmed BORSA isolates was evaluated against a panel including erythromycin (15 μg), fusidic acid (10 μg), clindamycin (2 μg), rifampin (5 μg), linezolid (30 μg), trimethoprim-sulfamethoxazole (1.25/23.75 μg), penicillin (10 units), and tetracycline (30 μg) (Bioanalyse, Türkiye).

### DNA Template Preparation

Cryopreserved isolates were revived on blood agar plates for 24 h prior to extraction. Genomic DNA was obtained using a boiling method, where approximately five colonies were suspended in 200 μL of nuclease-free water and incubated at 100 °C for 15 min. Following centrifugation to pellet cellular debris, the supernatant containing the DNA template was collected and stored at –80 °C for subsequent molecular analysis.

### Target Gene Detection

Molecular characterisation was performed by targeting the *nuc* and *mecA* genes. The *nuc* gene served as a species-specific marker for *S. aureus* confirmation and as an internal control for DNA template integrity. Concurrently, the *mecA* gene was evaluated to differentiate BORSA isolates (defined by the absence of *mecA*) from MRSA. All oligonucleotide primers were synthesised by Apical Scientific Sdn. Bhd., Malaysia. The specific primer sequences and expected amplicon sizes for the target gene *nuc* were: forward primer GCG ATT GAT GGT GAT ACG GT/3deoxyI/and reverse primer AGC CAA GCC TTG ACG AAC TAA AGC, with an expected amplicon size of 279 base pairs (14). Similarly, for the *mecA* gene, the forward and reverse primers were AAA ATC GAT GGT AAA GGT TGG C and AGT TCT GGA GTA CCG GAT TTG C, respectively. The expected amplicon size was 533 base pairs, as described by Lee (15). Thermal cycling parameters utilised for the PCR amplification of the *nuc* and *mecA* genes followed the reference work by Pournajaf et al. (16) and Karimzadeh et al. (17), respectively. For molecular quality control, *S. aureus* ATCC 25923 was utilised as the positive control for the *nuc* gene and as a negative control for the *mecA* gene. In the absence of a commercial MRSA reference strain, a well-characterised, in-house clinical MRSA isolate, previously validated by phenotypic disc diffusion and confirmed by *mecA* amplification, served as the *mecA* positive control. Sterilised nuclease-free water was included in every run as a no-template negative control.

Clinical and demographic data were retrospectively extracted from medical records using a standardised proforma. To ensure confidentiality and ethical compliance, all patient identifiers were anonymised and assigned unique alphanumeric research codes.

## Results

A total of 307 clinical isolates were subjected to susceptibility testing, including cefoxitin and oxacillin screening alongside the standard antibiotic panel. Blood cultures constituted the predominant specimen type. The complete distribution of clinical sources from which *S. aureus* was isolated is illustrated in Figure 2.

Reduced susceptibility to oxacillin by disc diffusion (inhibition zone ≤ 12 mm) was demonstrated by 13.4% (*n* = 41) of the isolates. Of these, 48.8% (*n* = 20) were confirmed to be resistant (MIC ≥ 2 μg/mL). Phenotypic characteristics (oxacillin susceptibility and β-lactamase status) of the study isolates from various specimen types are shown in Table 1.

The antibiogram distribution of all *S. aureus* isolates is shown in Figure 3. The isolates demonstrated universal susceptibility (100%) to cefoxitin, erythromycin, clindamycin, rifampin, linezolid, and trimethoprim-sulfamethoxazole. Conversely, resistance rates varied for other agents: fusidic acid and tetracycline were susceptible in 75% (*n* = 15) and 35% (*n* = 7) of the isolates, respectively. Notably, all isolates were categorised as penicillin-resistant. This classification was applied regardless of the initial MIC or zone diameter readings, necessitated by the confirmed production of β-lactamase as per CLSI M100 34th edition guidelines (10).

### Genotypic Characterisation of BORSA Isolates

All 20 isolates (100%) yielded positive amplification of the *nuc* gene, which confirmed the identification of *S. aureus*. Conversely, molecular screening for the methicillin resistance determinant, *mecA*, was uniformly negative across all samples. Representative agarose gel electrophoresis results for the *nuc* and *mecA* assays are presented in Figures 4a and 4b, respectively.

The demographic data of all 20 patients infected with BORSA were summarised in Table 2. Clinical characteristics and outcomes of patients with confirmed BORSA infections are summarised in Table 3. No statistically significant associations were identified between the evaluated clinical variables and patient outcomes. However, all deaths occurred among patients with bloodstream infections (100.0% vs. 53.3% in survivors; *P* = 0.114), while end-stage renal failure was more frequent among patients who died than among those who were discharged (60.0% vs. 26.7%; *P* = 0.290).

## Discussion

Accurate detection of BORSA remains a persistent diagnostic challenge that is insufficiently characterised in current literature. A critical limitation in routine screening is the potential for misclassification; BORSA strains frequently exhibit susceptibility to cefoxitin, the standard surrogate marker for methicillin resistance. Consequently, concurrent susceptibility testing using both cefoxitin and oxacillin is recommended to prevent these strains from evading detection (7). The reference standard for detecting BORSA isolates involves adding a β-lactamase inhibitor, such as clavulanic acid, to oxacillin MIC testing (18). This modification typically lowers the MIC by at least two-fold. However, this technique is not routinely performed in clinical laboratories, and most international guidelines lack a standardised method for BORSA detection. Therefore, the approach used in this study, which is screening via oxacillin disc diffusion followed by confirmation with gradient diffusion, serves as a valid alternative. The utilisation of these historical criteria was necessitated by the shift in current guidelines. This is because the current CLSI no longer recommends using the 1 μg oxacillin disc diffusion test for human *S. aureus* isolates, as it has been largely superseded by the highly reliable 30 μg cefoxitin disc diffusion screen or direct *mecA* gene testing.

In this study, the confirmed prevalence of BORSA among methicillin-susceptible *S. aureus* isolates was 6.5% (20/307). Notably, initial screening via oxacillin disc diffusion yielded a higher potential prevalence of 13.4% (41/307). However, upon confirmatory testing using gradient diffusion strips, only 48.8% (20/41) of these screened isolates met the definitive MIC criteria (≥ 2 μg/mL). This significant reduction highlights the limitations of the oxacillin disc diffusion method, which is known to exhibit higher rates of major and minor errors compared to MIC determination (18). The oxacillin disc diffusion method is a useful screening tool but is less reliable than MIC-based confirmation for borderline phenotypes such as BORSA. Disc diffusion interpretations are highly susceptible to small analytical variations (e.g., inoculum density, agar depth, incubation conditions, and subjective zone edge reading), which can shift zone diameters across interpretive breakpoints and result in minor/major categorical errors. This effect is amplified among isolates with oxacillin susceptibility near the breakpoint, leading to reduced positive predictive value of screening. In contrast, MIC determination provides a quantitative endpoint and is considered a more definitive approach for confirming reduced oxacillin susceptibility (18, 19). While our findings are limited to a single centre, this 6.5% prevalence rate falls well within the reported global range of < 1% to 12.5% (7, 20, 21).

A notable finding in this study was the predominance of BORSA isolates in bloodstream infections, with 65% (13/20) of cases representing bacteraemia. This contrasts with existing literature, where BORSA has typically been associated with skin and soft tissue infections, surgical wounds, respiratory tract infections, and urinary tract infections, often with a predilection for dermatology units (7). However, this high proportion is expected; as a tertiary care centre, our facility frequently manages highly acute cases where blood cultures are routinely prioritised. BORSA in hospital settings may cause bacteraemia. The high rate of primary bacteraemia observed in our setting suggests that the initial focus of infection is often clinically occult or unrecognised. This raises the question of endogenous sources; for instance, veterinary studies have reported BORSA colonisation rates of up to 14% in the nasal cavities of swine (22). A study has also shown that healthy healthcare personnel carry BORSA in their nasal cavities (23).

Phenotypic characterisation confirmed that 100% (20/20) of the identified BORSA isolates were β-lactamase positive, evidenced by both the nitrocefin disc test and the “keyhole phenomenon” observed in double-disc synergy assays. These findings align with global epidemiological data, which consistently report a high prevalence (75% to 80%) of β-lactamase production among clinical *S. aureus* isolates (24). The universal presence of this enzyme in our cohort is significant, as the hyperproduction of β-lactamase was the first documented cause of the BORSA phenotype (5). While staphylococcal β-lactamases hydrolyse natural penicillins with high efficiency, they also possess a residual capability to inactivate PRPs slowly. When synthesised in excessive quantities, this slow hydrolysis becomes sufficient to elevate oxacillin MICs to borderline levels, distinguishing these strains from standard MSSA.

The antibiogram profile of the 20 BORSA isolates revealed uniform susceptibility to non-beta- lactam agents, including erythromycin, clindamycin, rifampin, linezolid, and trimethoprim-sulfamethoxazole. Additionally, all isolates remained susceptible to cefoxitin, reinforcing their distinction from MRSA. Given the current absence of standardised international guidelines for BORSA management, therapeutic strategies must be guided by specific susceptibility patterns and the severity of clinical presentation (8). This broad susceptibility profile is clinically advantageous, as it offers viable therapeutic alternatives to glycopeptides, thereby supporting antimicrobial stewardship efforts to preserve vancomycin for MRSA infections. In our centre, the protocol prioritises the use of susceptible agents based on the antibiogram; however, clinical vigilance is paramount. In cases of therapeutic failure or persistent bacteraemia, escalation to vancomycin is mandated. Furthermore, early consultation with the Infectious Disease team is advocated to optimise regimen selection and ensure favourable patient outcomes.

Conversely, resistance rates to tetracycline (65%) and fusidic acid (25%) were notable in this cohort. As expected, all isolates were categorised as penicillin-resistant due to confirmed β-lactamase production. This finding is in accordance with the CLSI recommendation that penicillin susceptibility must not be inferred solely from zone diameters or MICs but requires phenotypic confirmation of enzyme activity. The universal penicillin resistance observed mirrors global trends (23) and highlights the ubiquity of the *blaZ* determinant in *S. aureus* populations. This resistance is mediated by the *blaZ* gene, which is frequently carried on mobile plasmids (25, 26), facilitating rapid horizontal transmission and maintenance within bacterial populations. The expression of *blaZ* is tightly regulated by a signal transduction system involving the *blaI* repressor and *blaR1* sensor. The presence of environmental β-lactams triggers the *blaR1* sensor, which subsequently cleaves the *blaI* repressor, inducing high-level enzyme production to neutralise the antibiotic threat.

Molecular analysis via PCR definitively confirmed the absence of the *mecA* gene across all 20 identified BORSA isolates. This genetic validation correlates perfectly with the phenotypic susceptibility to cefoxitin observed during initial screening. As cefoxitin is the standard surrogate marker for *mecA*-mediated resistance, this concordance reinforces that the borderline resistance observed in this cohort is not attributable to the classical PBP2a mechanism found in MRSA. These findings align with established consensus in the literature, which dictates that definitive classification of a strain as BORSA necessitates the exclusion of MRSA traits. Consequently, validation through PBP2a latex agglutination assays or molecular detection of *mec* determinants is considered the diagnostic gold standard (27). However, integrating these confirmatory tests into daily diagnostic workflows presents significant challenges. The substantial cost of molecular reagents, coupled with the requirement for specialised thermal cyclers and skilled technical personnel, often precludes adoption as standard operating procedures, particularly in resource-limited settings. As a result, many clinical laboratories may be forced to rely solely on phenotypic screening, which risks underestimating the true prevalence of this resistance phenotype.

The demographic analysis of the study cohort revealed a broad age distribution ranging from 25 to 78 years, with a mean age of 48.5 years. While the infection primarily affected adults, a single outlier involving a one-yearold infant indicates that BORSA can, albeit rarely, manifest in the paediatric population. The gender distribution was relatively balanced, affecting 11 males and no females. Of note, most BORSA cases had underlying metabolic and renal pathologies. Diabetes mellitus was the most common comorbidity (*n* = 13), followed by hypertension (*n* = 11) and dyslipidaemia (*n* = 5). Furthermore, 45% of the cohort (*n* = 9) suffered from chronic kidney disease or end-stage renal disease.

Although no statistically significant associations were identified between the evaluated clinical characteristics and patient outcomes, these findings should be interpreted cautiously because the small sample size (*n* = 20) may have limited the statistical power to detect true differences. Nevertheless, all deaths occurred among patients with bloodstream infections, and end-stage renal failure was more frequent among non-survivors (60.0% vs. 26.7%). Although these associations were not statistically significant, the observed patterns suggest that invasive infection and underlying renal impairment may contribute to poorer outcomes and warrant further investigation in larger studies.

While literature specifically characterising risk factors for BORSA remains scarce, the comorbidity profile observed in this study mirrors established epidemiological patterns for general *S. aureus* infections. Previous studies have consistently identified diabetes mellitus, chronic haemodialysis, and dermatological conditions as major predisposing factors (28). This susceptibility is likely multifactorial; patients with diabetes mellitus and renal failure exhibit significantly higher rates of nasal and cutaneous *S. aureus* colonisation, creating a persistent endogenous reservoir for potential infection (29). Moreover, the management of these chronic conditions often necessitates frequent invasive procedures, such as intravascular catheterisation for haemodialysis. These interventions breach the skin’s mechanical barriers, providing a direct portal of entry for invasive staphylococcal disease (30). Given these observations, larger comparative studies would help determine whether the risk profile for BORSA differs distinctively from MSSA or MRSA. Identifying specific high-risk cohorts would allow for more targeted surveillance strategies and the prompt initiation of appropriate empiric therapy.

Ten patients were managed with vancomycin, an agent typically reserved for severe BORSA infections refractory to first-line therapies such as cloxacillin. While vancomycin is frequently employed in this context, its use is not currently supported by official, evidence-based consensus guidelines (30). Notably, a mortality rate of 50% (*n* = 5) was observed within this cohort. However, given the retrospective nature of this review, it is difficult to attribute this poor outcome solely to therapeutic failure; confounding variables, particularly the baseline severity of the infection, likely contributed to the high mortality. Conversely, nine patients achieved clinical resolution following treatment with β-lactams or β-lactam/β-lactamase inhibitor combinations and were successfully discharged. This aligns with previous findings suggesting that β-lactams remain a viable therapeutic option for infections of lower severity, even in the presence of elevated MICs (31). Due to the absence of standardised protocols for BORSA bacteraemia, therapeutic strategies currently rely on case-by-case clinical judgement, resulting in variable antimicrobial regimens and outcomes (32).

A further complication is the lack of a defined treatment duration. While uncomplicated MSSA bacteraemia is standardly treated for 14 days following culture clearance (33), it remains unclear whether this protocol is sufficient for BORSA. Future research is urgently required to delineate the optimal clinical management and duration for these infections. Moving forward, routine screening for oxacillin MICs ≥ 2 μg/mL is advisable. In such cases, particularly those involving complicated or recalcitrant infections, clinicians should consider further susceptibility testing and potential modification of the antimicrobial regimen.

### Limitations

Prevalence rates and antibiotic susceptibility patterns were analysed only from one centre. The local antibiogram is inevitably influenced by the specific prescribing practices, patient demographics, and infection control measures unique to this tertiary referral centre. The study did not screen for the *mecB* and *mecC* genes, which are rare and divergent homologs that can also confer methicillin resistance. Consequently, there remains a theoretical possibility that a small subset of *mecA*-negative, oxacillin-resistant isolates classified here as BORSA could potentially be *mecB*- or *mecC*-MRSA. The study did not employ gene sequencing to confirm the specific genetic mechanisms driving resistance, such as point mutations in PBPs or specific *blaZ* gene variants. It is also noted that MICs were determined using gradient diffusion strips rather than broth microdilution, the latter being the gold standard reference method, which may introduce minor variances in MIC precision near resistance breakpoints. Further studies incorporating a non-BORSA control group are recommended to determine if underlying comorbidities are statistically significant risk factors for BORSA infection.

## Conclusion

This study confirms the presence of BORSA isolates within the clinical setting of our hospital. The findings suggest that hyperproduction of β-lactamases may drive the resistance mechanism in these local strains. Misidentifying BORSA as MRSA may lead to the unnecessary use of last-line agents such as vancomycin, whereas misclassifying it as susceptible MSSA could result in therapeutic failure with standard penicillins. Therefore, this study underscores the necessity for clinical microbiology laboratories to remain vigilant for *S. aureus* strains with borderline oxacillin resistance patterns and to consider implementing supplementary testing methods, such as oxacillin MIC determination and β-lactamase hyperproduction screening, to guide appropriate antimicrobial stewardship and ensure optimal patient outcomes.

## Figures and Tables

**Figure 1:**
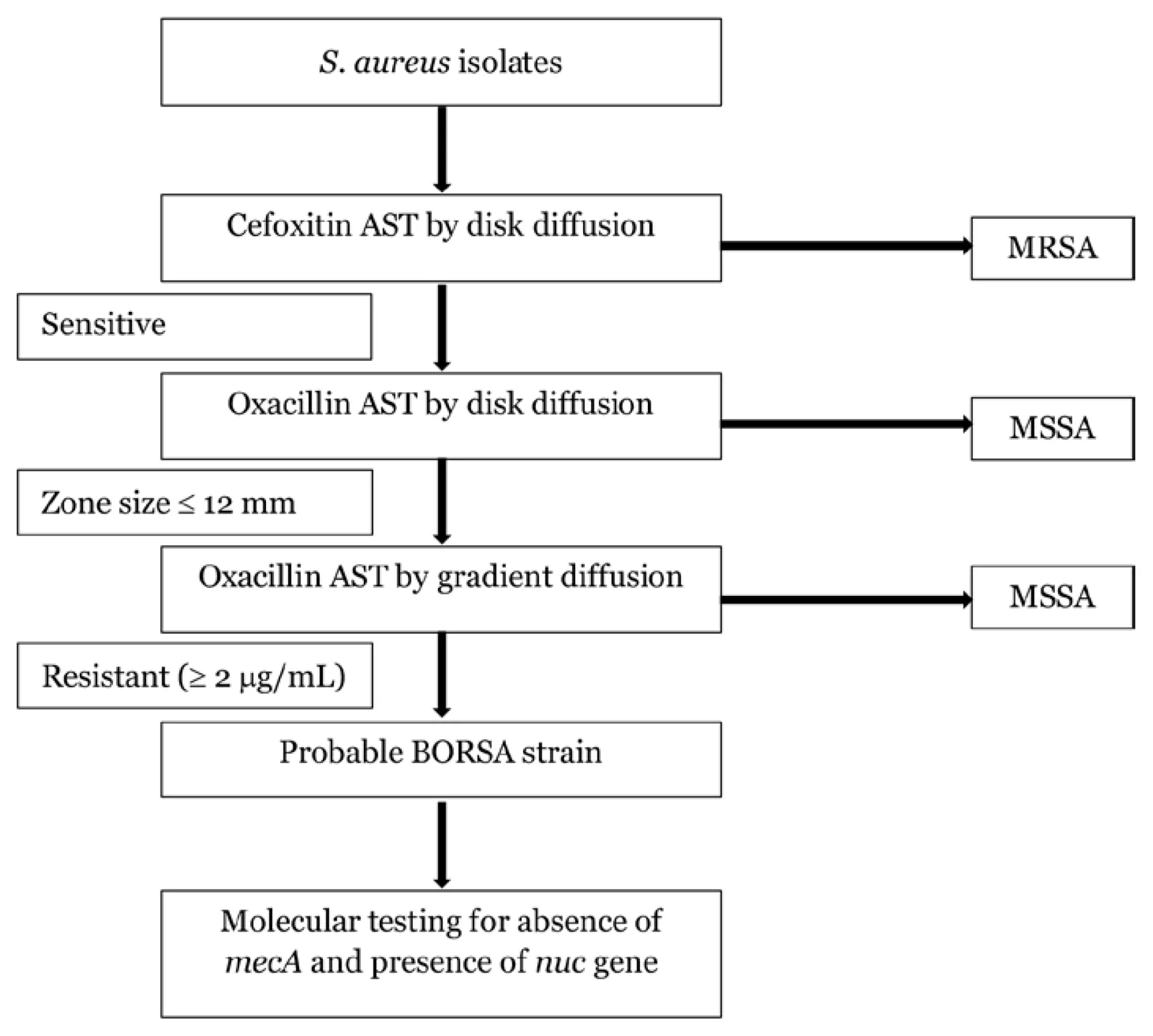
Flowchart detailing the laboratory workflow for the screening, MIC determination, and molecular confirmation of borderline oxacillin-susceptible *S. aureus* (BORSA) isolates. AST = antibiotic susceptibility testing; MRSA = methicillin-resistant *S. aureus*; MSSA = methicillin-sensitive *S. aureus*.

**Figure 2:**
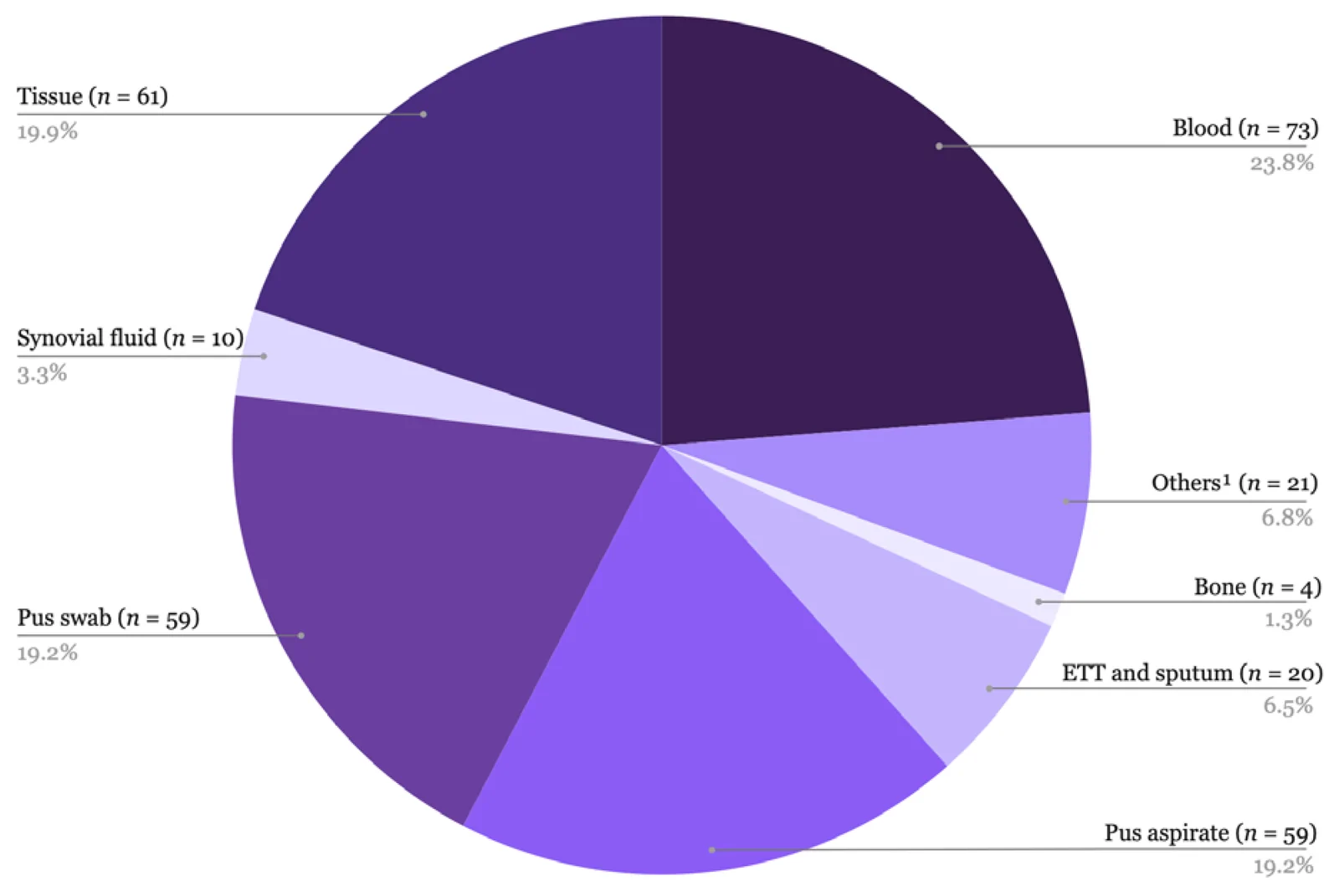
Distribution of *S. aureus* isolates categorised by clinical specimen type (*n* = 307). ^1^Other samples included are bronchoalveolar lavage, ear and eye swabs, fine needle aspirate, peritoneal, dialysate, and pleural fluids, high vaginal swab, urine, cephalic vein, and high vaginal swab.

**Figure 3:**
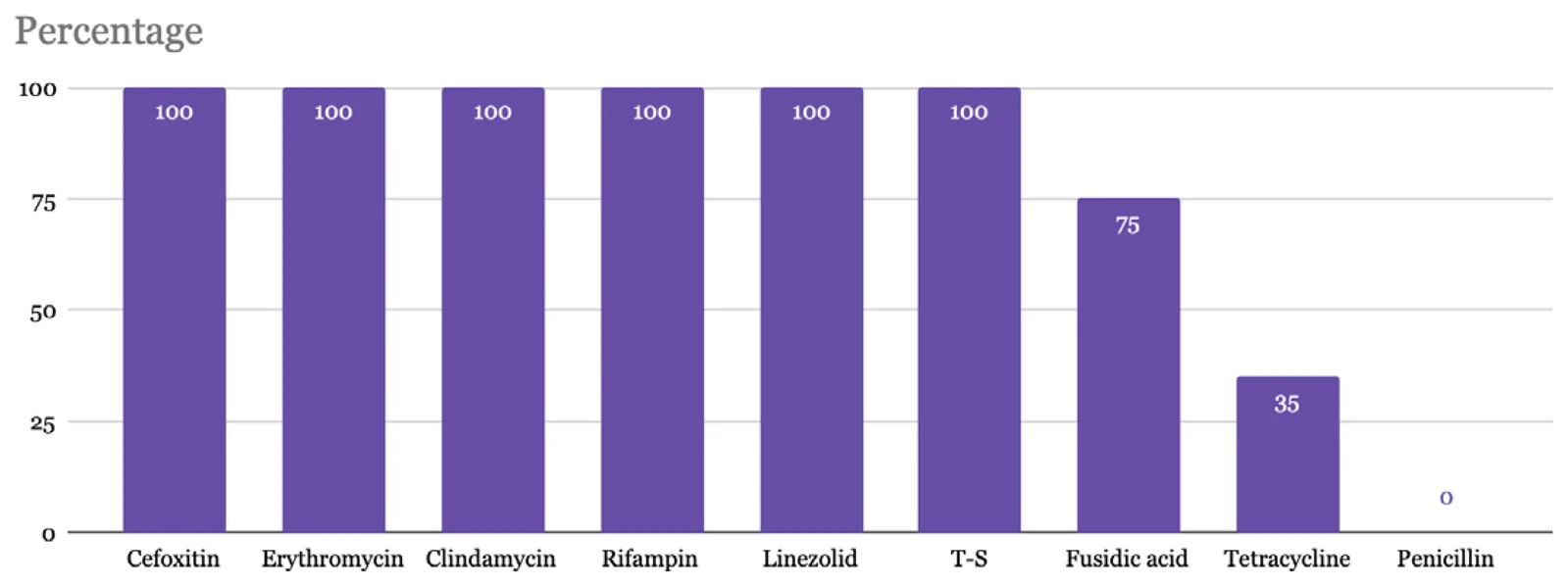
Antimicrobial susceptibility profile of BORSA isolates. The bar chart depicts the susceptibility rates (*n* = 20) across the tested antibiotic panel. Note that penicillin susceptibility is reported at 0% due to β-lactamase production. T-S = trimethoprim-sulfamethoxazole.

**Figure 4a:**
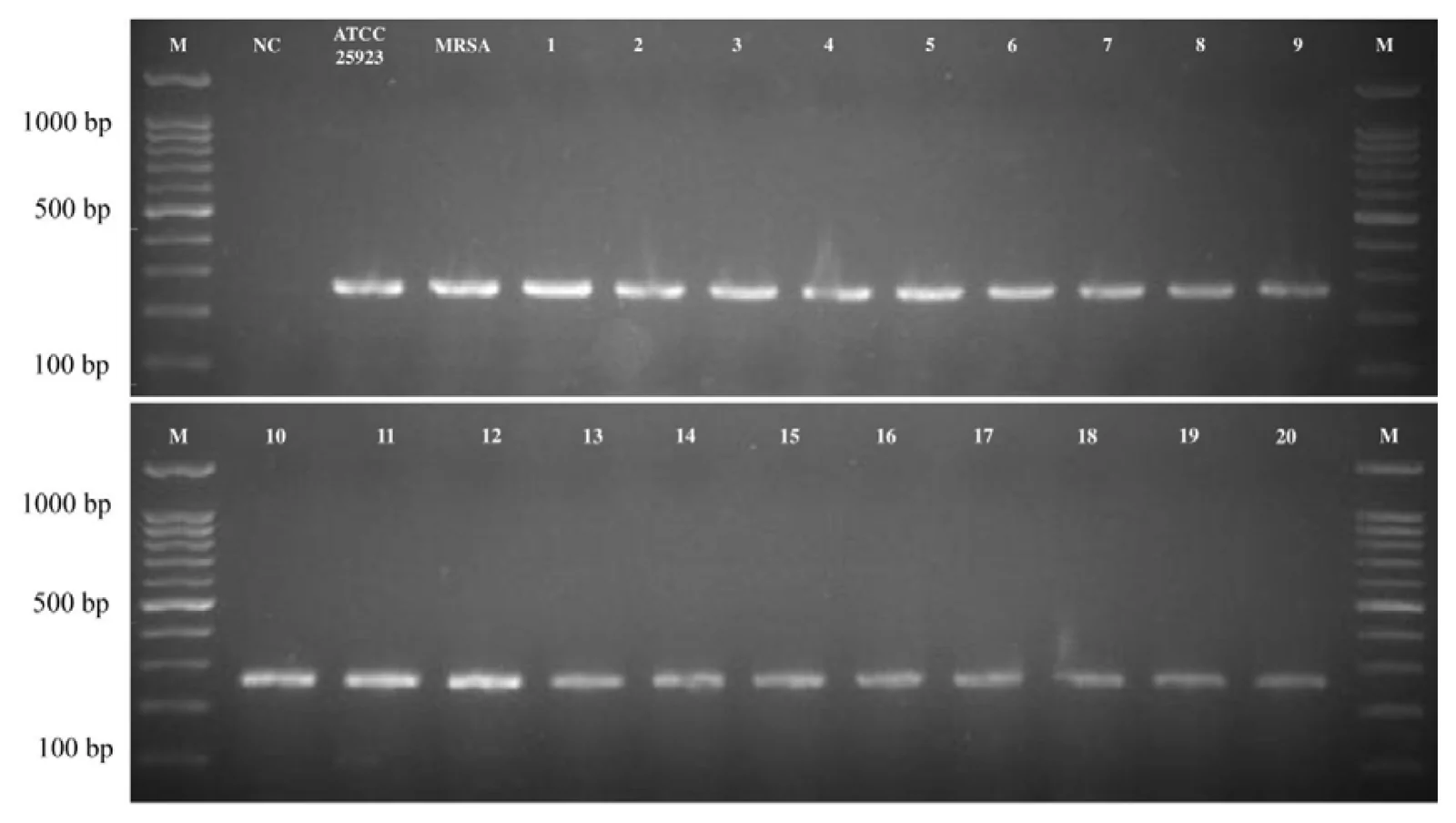
PCR amplification of the *nuc* gene. The presence of the specific amplicon band confirms the identity of the isolates as *S. aureus*. Lane M: 100 bp DNA ladder; ATCC 25923: Positive control (*S. aureus* ATCC 25923); NC: Negative control (PCR water); MRSA: Positive control (MRSA from clinical sample); Lanes 1–20: Clinical isolates showing positive amplification.

**Figure 4b:**
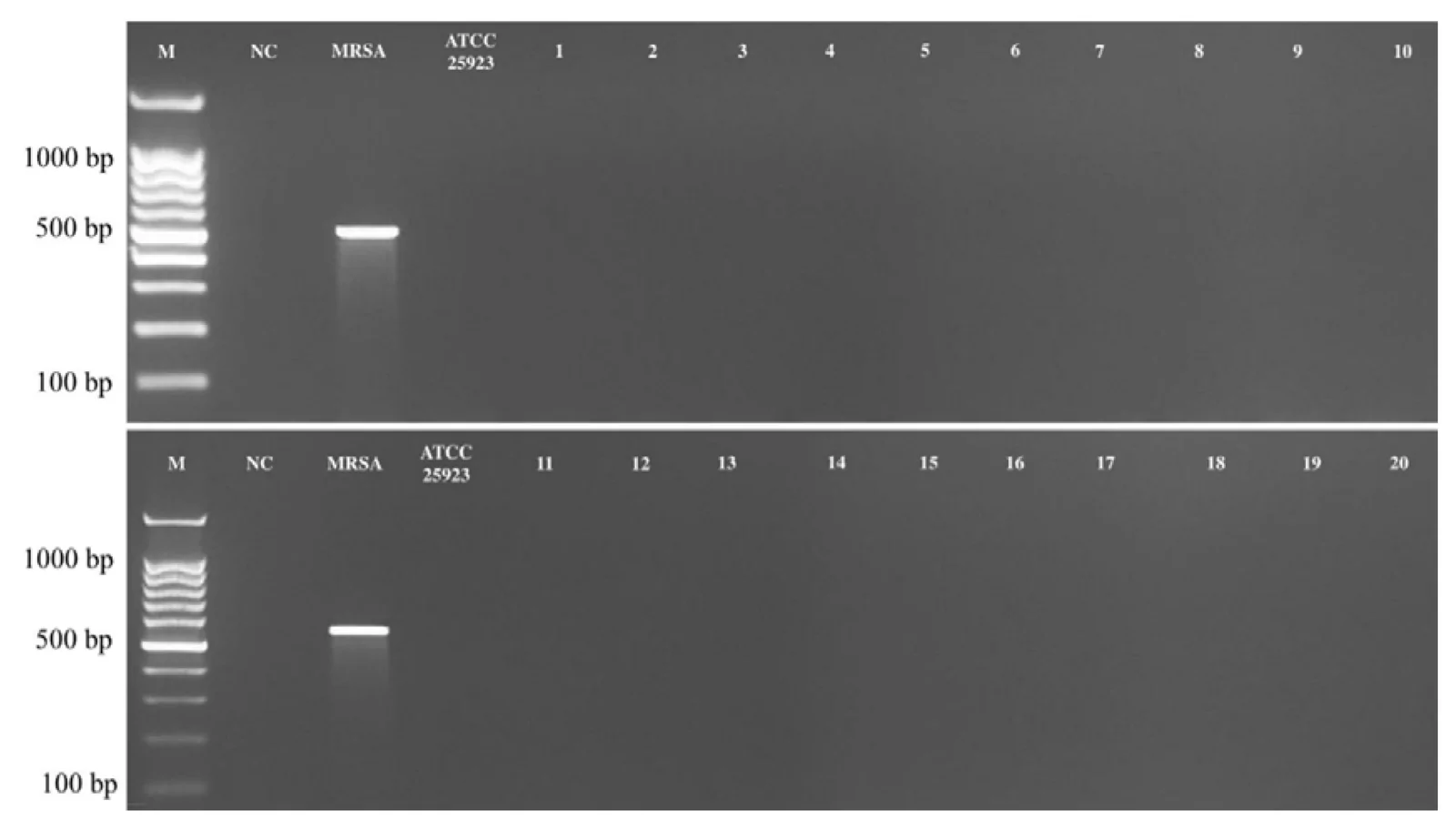
PCR screening for the *mecA* gene. Gel electrophoresis results demonstrating the absence of *mecA* amplification in the study isolates, confirming their status as non- MRSA (*mecA*-negative). Lane M: 100 bp DNA ladder; ATCC 25923: Negative control (*S. aureus* ATCC 25923); NC: Negative control (PCR water); MRSA: Positive control (MRSA from clinical sample); Lanes 1 to 20: Clinical isolates (negative for *mecA*).

**Table 1:** Phenotypic characteristics (oxacillin susceptibility and β-lactamase status) of the study isolates (*n* = 20).

Isolate no.	Specimen type	Oxacillin (screening, mm)	Oxacillin MIC (gradient diffusion)	Nitrocefin	Double-disc synergy
1	Swab	9	2	Positive	Positive
2	Blood	≥ 4	2	Positive	Positive
3	Blood	≥ 4	3	Positive	Positive
4	Blood	≥ 4	3	Positive	Positive
5	Blood	≥ 4	2	Positive	Positive
6	Blood	≥ 4	2	Positive	Positive
7	Blood	≥ 4	2	Positive	Positive
8	Blood	≥ 4	3	Positive	Positive
9	Blood	≥ 4	3	Positive	Positive
10	Pus	8	2	Positive	Positive
11	Pus swab	10	3	Positive	Positive
12	Peritoneal dialysate fluid	9	2	Positive	Positive
13	Blood	≥ 4	3	Positive	Positive
14	Pus aspirate	6	2	Positive	Positive
15	Blood	≥ 4	3	Positive	Positive
16	Blood	≥ 4	2	Positive	Positive
17	Blood	≥ 4	2	Positive	Positive
18	Tissue	9	2	Positive	Positive
19	Blood	≥ 4	3	Positive	Positive
20	Pus swab	9	2	Positive	Positive

**Table 2:** Demographic, clinical characteristics, antibiotics used, and outcomes of patients infected with confirmed BORSA strains (*n* = 20).

Isolate no.	Sample type	Age, gender	Co-morbid(s)	Days of hospitalisation	Antibiotic(s)	Outcome
1	Swab	61, M	Valvular heart disease	25	Cloxacillin, then discharged with ampicillin-sulbactam	Discharged
2	Blood	54, M	DM/ HTN/ IHD	6	Piperacillin-tazobactam, discharged with trimethoprim- sulfamethoxazole	Discharged
3	Blood	58, M	DM/ HTN/ HLP/ ESRF/ IHD	13	Vancomycin	Died
4	Blood	44, M	Asthma/ Hepatitis B	17	Vancomycin	Died
5	Blood	36, F	ESRF	10	Vancomycin	Died
6	Blood	58, M	DM/ ESRF/ Hepatitis B	12	Cloxacillin, then vancomycin for 3 days prior to death	Died
7	Blood	39, M	DM/ HTN/ HLP/ ESRF/	14	Vancomycin	Discharged
8	Blood	69, M	DM/ HTN	43	Vancomycin, then piperacillin-tazobactam	Died
9	Blood	59, F	DM/ HTN/ CRD	7	Ampicillin-sulbactam, then discharged with cloxacillin	Discharged
10	Pus	66, M	DM/ HTN/ CRD	12	Cloxacillin	Discharged
11	Swab	51, M	DM	Outpatient	Cloxacillin	Discharged
12	PD fluid	28, F	DM/ HTN/ ESRF	10	Cloxacillin	Discharged
13	Blood	76, M	DM/ HTN/ HLP/	45	Vancomycin	Discharged
14	Pus	53, F	HTN/ HLP/ ESRF	5	Ampicillin-sulbactam	Discharged
15	Blood	64, F	DM/ HTN/ HLP/	22	Vancomycin	Discharged
16	Blood	1, F	Heart block	10	Vancomycin	Discharged
17	Blood	25, M	Eczema	17	Vancomycin	Discharged
18	Tissue	27, F	None	4	Ampicillin-sulbactam	Discharged
19	Blood	65, F	DM/ HTN/ HLP/ ESRF/	Not hospitalised	No information	Discharged
20	Swab	37, F	DM	13	Ampicillin-sulbactam	Discharged

M = male; F = female; DM = diabetes mellitus; HTN = hypertension; HLP = hyperlipidaemia; ESRF = end-stage renal failure;

CRD = chronic renal disease; IHD = ischaemic heart disease

**Table 3:** Clinical characteristics, comorbidities and outcomes of patients infected with confirmed BORSA strains (*n* = 20).

Variables		Discharged (*n* = 15) *n* (%)	Died (*n* = 5) *n* (%)	Overall (*n* = 20) *n* (%)	*P-value (Fisher’s exact test)
Gender	Male	7 (46.7)	4 (80.0)	11 (55.0)	0.319
	Female	8 (53.3)	1 (20.0)	9 (45.0)	

Sample type	Blood	8 (53.3)	5 (100.0)	13 (65.0)	0.114
	Non-blood	7 (46.7)	0 (0.0)	7 (35.0)	

Diabetes mellitus	No	5 (33.3)	2 (40.0)	7 (35.0)	1.000
	Yes	10 (66.7)	3 (60.0)	13 (65.0)	

Hypertension	No	6 (40.0)	3 (60.0)	9 (45.0)	0.617
	Yes	9 (60.0)	2 (40.0)	11 (55.0)	

Hyperlipidaemia	No	11 (73.3)	4 (80.0)	15 (75.0)	1.000
	Yes	4 (26.7)	1 (20.0)	5 (25.0)	

Chronic renal disease	No	13 (86.7)	5 (100.0)	18 (90.0)	1.000
	Yes	2 (13.3)	0 (0.0)	2 (10.0)	

End-stage renal failure	No	11 (73.3)	2 (40.0)	13 (65.0)	0.290
	Yes	4 (26.7)	3 (60.0)	7 (35.0)	

Ischaemic heart disease	No	14 (93.3)	4 (80.0)	18 (90.0)	0.447
	Yes	1 (6.7)	1 (20.0)	2 (10.0)	
